# *Trichomonas vaginalis* Induces SiHa Cell Apoptosis by NF-*κ*B Inactivation via Reactive Oxygen Species

**DOI:** 10.1155/2017/3904870

**Published:** 2017-12-18

**Authors:** Juan-Hua Quan, Byung-Hun Kang, Jung-Bo Yang, Yun-Ee Rhee, Heung-Tae Noh, In-Wook Choi, Guang-Ho Cha, Jae-Min Yuk, Young-Ha Lee

**Affiliations:** ^1^Department of Gastroenterology, Affiliated Hospital of Guangdong Medical University, Zhanjiang, Guangdong Province 524001, China; ^2^Department of Obstetrics and Gynecology, Chungnam National University School of Medicine, Daejeon 35015, Republic of Korea; ^3^Department of Infection Biology, Chungnam National University School of Medicine, Daejeon 35015, Republic of Korea

## Abstract

*Trichomonas vaginalis *induces apoptosis in host cells through various mechanisms; however, little is known about the relationship between apoptosis, reactive oxygen species (ROS), and NF-*κ*B signaling pathways in the cervical mucosal epithelium. Here, we evaluated apoptotic events, ROS production, and NF-*κ*B activity in* T. vaginalis*-treated cervical mucosal epithelial SiHa cells, with or without specific inhibitors, using fluorescence microscopy, DNA fragmentation assays, subcellular fractionation, western blotting, and luciferase reporter assay. SiHa cells treated with live* T. vaginalis* at a multiplicity of infection of 5 (MOI 5) for 4 h produced intracellular and mitochondrial ROS in a parasite-load-dependent manner. Incubation with* T*.* vaginalis* caused DNA fragmentation, cleavage of caspase 3 and PARP, and release of cytochrome* c* into the cytoplasm.* T. vaginalis*-treated SiHa cells showed transient early NF-*κ*B p65 nuclear translocation, which dramatically dropped at 4 h after treatment. Suppression of NF-*κ*B activity was dependent on parasite burden. However, treatment with the ROS scavenger, N-acetyl-C-cysteine (NAC), reversed the effect of* T*.* vaginalis *on apoptosis and NF-*κ*B inactivation in SiHa cells. Taken together,* T*.* vaginalis *induces apoptosis in human cervical mucosal epithelial cells by parasite-dose-dependent ROS production through an NF-*κ*B-regulated, mitochondria-mediated pathway.

## 1. Introduction

*Trichomonas vaginalis, *a flagellated lumen-dwelling protozoan parasite, causes a sexually transmitted disease, with an estimated 275 million new cases worldwide each year [1]. This parasite causes vaginitis and cervicitis in women and asymptomatic urethritis and prostatitis in men, with associated morbidities including endometritis, preterm birth, and viral infections [2, 3]. Epidemiological studies have also shown that* T. vaginalis* acts as a cofactor in transmitting human immunodeficiency virus (HIV) type 1, and infection of women with* T. vaginalis* increases the risk of HIV infection [4]. However, the pathophysiology of trichomoniasis is not well established.

Apoptosis, a biological event induced by the activation of a series of enzymes known as caspases, may occur when a cell is damaged beyond repair, infected with a pathogen, or stressed due to DNA damage or toxic chemicals. Studies using* in vitro* models have shown that* T. vaginalis* induces apoptosis of various types of cells, including a murine monocyte/macrophage cell line [5–7], human monocyte-derived macrophages [8, 9], primary human vaginal epithelial cells [10, 11], human cervical cancer cells (SiHa cells), and vaginal epithelial cells (MS74 cells) [12]. Although there are some reports of apoptosis induced by* T. vaginalis*, the mechanisms of apoptosis in human cervical mucosal epithelial cells infected with* T. vaginalis* are not well elucidated.

NF-*κ*B is an essential transcription factor that regulates diverse physiological processes, including cell growth, differentiation, inflammatory responses, and apoptosis [13]. In normal conditions, NF-*κ*B remains in an inactive form in the cytoplasm in association with the inhibitory family of I*κ*B molecules. Upon stimulation, I*κ*B-*α* is phosphorylated by I*κ*B kinase (IKK) and degraded, leading to nuclear translocation of NF-*κ*B p65 and p50 to induce the expression of various genes controlling inflammatory and immune responses [14]. Plumbagin has been shown to inhibit cell growth and potentiate apoptosis in human GC cells through the NF-*κ*B pathway [15].* T*.* vaginalis* inhibits proinflammatory cytokine production in macrophages by suppressing NF-*κ*B activation [16]. However, there are no data on the role of NF-*κ*B signaling pathways in cervical epithelial SiHa cells in response to* T. vaginalis*.

The cervicovaginal mucosa is the first line of defense against pathogenic organisms. Reactive oxygen species (ROS) are essential for many biological functions including apoptosis and NF-*κ*B activation and are known for their ability to regulate certain essential signaling pathways. Their targets include protein kinase B (Akt), nuclear factor kappa-light-chain enhancer of activated B cells (NF-*κ*B), and mitogen-activated protein kinases (MAPKs) [17]. ROS also function as intermediates in cellular processes such as inflammatory responses, cell cycle progression, apoptosis, aging, and cancer [17, 18]. Previous reports have shown that dioscin (a natural product) and casticin (one of the main components of* Fructus viticis*) induce apoptosis of HeLa and SiHa cells by promoting ROS-mediated DNA damage and the mitochondrial signaling pathway [19, 20]. Activation of MAPK is required for ROS generation and exocytosis in HMC-1 cells by* T. vaginalis*-derived secretory products [21].* T. vaginalis* induces IL-1*β* production in human prostate epithelium through activation of ROS [22]. There are numerous reports of ROS production or ROS function in various cell lines or in response to stimulants. However, the effect of ROS in* T*.* vaginalis*-treated cervical mucosal epithelial cells has not been described to date. Specifically, the roles of ROS in relation to the NK-*κ*B signaling pathway for apoptosis induction in* T*.* vaginalis*-treated cervical mucosal epithelial cells are unclear. Thus, to elucidate this mechanism, human cervical mucosal epithelial SiHa cells were treated with* T. vaginalis*, and apoptotic features, ROS production, and NF-*κ*B activity were evaluated using fluorescence microscopy, DNA fragmentation assays, subcellular fractionation, western blotting, and luciferase assays.

## 2. Materials and Methods

### 2.1. Antibodies and Reagents

Antibodies to poly-(ADP-ribose) polymerase (PARP), caspase 3, cleaved caspase 3, cytochrome* c*, NF-*κ*B p65, Bcl-2, COX IV, Histone H3, and *β*-actin were purchased from Cell Signaling Technology (Danvers, MA, USA). Mouse monoclonal anti-*α*-tubulin was purchased from Santa Cruz Biotechnology (Santa Cruz, CA, USA). N-Acetyl-L-cysteine (NAC), dihydroethidium (DHE), MitoSOX™ Red, 3-(4,5-dimethyl-2-thiazolyl)-2,5-diphenyl-2H-tetrazolium bromide (MTT), and staurosporine (STS) were purchased from Sigma-Aldrich Chemical Co. (St. Louis, MO, USA).

### 2.2. Live* T. vaginalis* Culture

The T016 strain of* T. vaginalis* was cultured in a glass, screw-capped tube containing Diamond's trypticase-yeast extract-maltose (TYM) medium (NAPCO, Winchester, VA, USA) supplemented with 10% heat-inactivated horse serum (Sigma-Aldrich) in 5% CO_2_ at 37°C for 24 h [12]. Cultured parasites were monitored for motility and the viability of* T. vaginalis *was determined before each experiment using trypan blue staining (>99%).

### 2.3. Preparation of* T. vaginalis* Excretory/Secretory Product (ESP)

*T. vaginalis* ESP were prepared as described previously [12]. To prepare the* T. vaginalis* ESP, freshly purified trophozoites (1 × 10^7^ cells/mL) were incubated with TYM medium at 37°C for 1 h in 5% CO_2_. After centrifugation for 30 min at 10,000*g*, the ESP-containing supernatant was filtered through a 0.2 *μ*m pore filter and stored at −70°C. The* T. vaginalis* ESP concentrations were determined by the Bradford assay with bovine serum albumin (BSA) as the standard.

### 2.4. Culture of SiHa Cells

The human cervical mucosal epithelial cancer cell line, SiHa, was obtained from the American Type Culture Collection (ATCC, Manassas, VA, USA) and maintained in Dulbecco's Modified Eagle's Medium (DMEM) supplemented with 10% heat-inactivated fetal bovine serum (FBS; Gibco BRL, Grand Island, NY, USA) and antibiotic-antimycotic solution (Gibco BRL) in a 5% CO_2_ atmosphere at 37°C.

### 2.5. Study Design

SiHa cells were seeded on 96-well plates (for MTT assay), 12-well cover-slips (for ROS detection), or 100 mm culture dishes (for western blotting, DNA fragmentation assay, and transfection) at various concentrations and grown to confluence at 37°C in 5% CO_2_.

We first evaluated ROS production in* T*.* vaginalis*-treated SiHa cells by fluorescence microscopy using DHE and MitoSox. Next, we investigated the induction of apoptosis in SiHa cells after* T. vaginalis* treatment by DNA fragmentation assay and western blotting. Also, NF-*κ*B activity was determined in* T. vaginalis*-treated cells by western blotting and luciferase reporter assay. To investigate the role of ROS in inducing apoptosis,* T. vaginalis*-treated SiHa cells were pretreated with a selective ROS scavenger, NAC, for 30 min, and then evaluated for apoptotic features and NF-*κ*B activity. The control group was just SiHa cell not treated with* T. vaginalis *or* T. vaginalis *ESP. Each experiment was performed at least three times.

### 2.6. Cell Viability Assay

Cell viability was determined using an MTT assay. Briefly, cells were seeded at a density of 5 × 10^3^ cells/well in 96-well plates. The plates were incubated at 37°C for 24 h to adhere the SiHa cells to the bottom of the well. Different concentrations of NAC (0.1, 1, 10 mM) were then added to each well of 96-well plates for 24 h. Then, 10 *μ*L of MTT solution (5 mg/mL) was added and cells were further incubated at 37°C for 4 h. Following the removal of MTT solution, water-insoluble formazan was dissolved by adding 100 *μ*L of dimethyl sulfoxide (DMSO) to each well. Finally, optical density (OD) was measured at 570 nm using a microplate reader (Sunnyvale, CA, USA). Data were shown as the absorbance relative to the untreated control.

### 2.7. DNA Fragmentation

DNA was isolated from 1 × 10^6^ SiHa cells using a genomic DNA extraction kit (iNtRON Biotechnology, Seoul, Korea). An equal amount of DNA was loaded into each well of 2% agarose gels containing ethidium bromide (0.5 *μ*g/mL) and separated electrophoretically using Tris-borate-EDTA (pH 8.0) as the running buffer (89 mM Tris-borate, 2 mM EDTA). Migrating DNA bands were visualized with a UV transilluminator (Gel Doc, Bio-Rad Laboratories, Ltd., Hercules, CA, USA).

### 2.8. Measurement of ROS Generation

*(1) DHE. *Intracellular ROS generation was detected by fluorescence microscopy using DHE. SiHa cells (1 × 10^5^ cells/well; 12-well plate) seeded on a cover-slip were cultured in DMEM supplemented with 10% FBS and the culture medium was replaced when cells reached 80% confluence. To evaluate the generation of ROS, SiHa cells were treated with live* T. vaginalis *(MOI 1 and 5) and* T. vaginalis* excretory/secretory product (ESP, 20 and 100 *μ*g/mL) for 0.5 h, 1 h, and 4 h. After incubation, culture supernatants were removed and cells were washed twice with 1× PBS before incubating with 10 *μ*M DHE in fresh medium, free of FBS, at 37°C for 30 min in the dark. After washing with HBSS buffer, intracellular ROS, as indicated by DHE fluorescence, was detected with a fluorescence microscope (Olympus BX51, Japan).

*(2) Mitochondrial Superoxide Detection.* Mitochondrial superoxide formation was detected by fluorescence microscopy using MitoSOX Red as a specific fluorescent probe. SiHa cells (1 × 10^5^ cells/well; 12-well plate) seeded on a cover-slip were cultured in DMEM supplemented with 10% FBS and the culture medium was replaced when the cells reached 80% confluence. To evaluate the generation of ROS, SiHa cells were treated with live* T. vaginalis *(MOI 1 and 5) and* T. vaginalis* ESP (20 and 100 *μ*g/mL) for 0.5 h, 1 h, and 4 h. After incubation, culture supernatants were removed and cells were washed twice with 1× PBS. MitoSOX staining was performed according to the manufacturer's protocol. Briefly, cells were incubated with 1 mL of 5 *μ*M of the MitoSOX reagent probe in the dark (covered with foil) and placed at 37°C in a humidified incubator with 5% CO_2_ for 10 min. After incubation, cells were washed thoroughly with warm PBS and mounted for imaging. MitoSOX Red was visualized using a fluorescence microscope (Olympus BX51). All experiments were performed on triplicate samples and fluorescence intensity was calculated using ImageJ software, and graph was plotted using SigmaPlot 12.5 (Systat Software, San Jose, CA).

### 2.9. Subcellular Fractionation

Isolation of cytosol, mitochondrial, and nuclear subcellular fractions were performed as described previously with minor modifications [23]. Cytosolic extracts free of nuclei and mitochondria were prepared as follows. Briefly, cells were washed in ice-cold PBS (pH 7.2) and then in hypotonic extraction buffer (HEB; 50 mM PIPES, 50 mM KCl, 5 mM EGTA, 2 mM MgCl_2_, 1 mM dithiothreitol, 0.1 mM PMSF, pH 7.4) and harvested by centrifugation. Pellets were resuspended in HEB and lysed in a Dounce homogenizer. These cell lysates were then centrifuged at 100,000*g* for 60 min at 4°C and the supernatants were flash-frozen in cold ethanol and stored in aliquots at −80°C.

Nuclear and mitochondrial fractions were prepared by washing cells in ice-cold PBS and then resuspending in an isotonic homogenization buffer (10 mM Tris–HCl, pH 7.5, 0.25 M sucrose, 10 mM KCl, 1 mM EDTA, 1 mM dithiothreitol, 0.1 mM phenylmethylsulphonyl fluoride, EDTA-free complete cocktail of protease inhibitors [Roche, Switzerland]). After 60 strokes in a Dounce homogenizer, the unbroken cells were removed by centrifugation at 30*g* for 10 min. The nuclei were pelleted by centrifugation at 1,000*g* for 10 min and supernatants were centrifuged at 14,000*g* for 20 min. The supernatants were collected as the mitochondrial fraction. The quality of the fraction experiments was confirmed by assessing the presence of *α*-Tubulin, COX IV, and Histone H3 for cytosol, mitochondrial, and nuclear fraction, respectively.

### 2.10. Western Blotting

Equal amounts of fractioned proteins were separated by SDS-PAGE and transferred to a polyvinylidene difluoride membrane. The membranes were blocked in Tris-buffered saline (20 mM Tris, 137 mM NaCl, pH 7.6) containing 0.1% Tween-20 (TBST) and 5% skim milk. After being washed once in TBST, membranes were incubated overnight at 4°C with the primary antibodies diluted in TBST supplemented with 5% BSA. The antibodies used were as follows: anti-caspase 3, anti-cleaved caspase 3, anti-PARP, anti-NF-*κ*B p65, anti-Bcl-2, and anti-cytochrome* c*. Following three consecutive washes in TBST, membranes were incubated for 2 h with horseradish peroxidase-conjugated anti-mouse or anti-rabbit IgG (Santa Cruz Biotechnology) diluted 1 : 10,000 with incubation buffer, as described above. After extensive washing, bound secondary antibodies were visualized using an enhanced ECL chemiluminescence detection kit (GE Healthcare, Little Chalfont, UK).

### 2.11. Transfection and Luciferase Reporter Assay

The pGL3-NF-*κ*B promoter constructs (kindly provided by Dr. Kyung-Cheol Sohn, Department of Dermatology, Chungnam National University) were transfected into SiHa cells with LipofectAmine 2000 (Invitrogen, Carlsbad, California, USA). Luciferase activity was assayed with a luciferase assay kit (Promega, Madison, WI, USA) described as follows. SiHa cells (1 × 10^6^) were plated on 60 mm Petri dishes. After overnight incubation, cells were transfected with an NF-*κ*B reporter plasmid linked to a luciferase gene and kept for 48 h, after which the medium was changed. Cells were treated with* T. vaginalis* for 2 h. Cell extracts were then prepared in 500 *μ*L of 1× reporter lysis buffer. Lysates were centrifuged at 13,000*g* for 5 min and luciferase activity was measured using a Microlumat LB96P Luminometer (Perkin Elmer, Wallac, Inc., USA). Luciferase activity was normalized relative to *β*-galactosidase activity in each sample.

### 2.12. Statistical Analysis

Data were presented as means ± SD. Statistical significance was determined by ANOVA (StatView; Abacus Concepts Inc., Berkeley, CA, USA). All experiments were performed at least in triplicate on separate days. Differences were considered significant at *P* values < 0.05.

## 3. Results

### 3.1. *T*.* vaginalis* Led to the Generation of Intracellular and Mitochondrial ROS in SiHa Cells, in Parasite-Burden-Dependent Manner

Oxidative stress is an important factor for toxicity associated with apoptosis.* T*.* vaginalis* resides in the vagina and colonizes the cervix. It attaches to human host epithelial cells to establish and maintain adhesion. To investigate whether ROS is generated in cervical mucosal epithelial SiHa cells after* T*.* vaginalis *treatment, intracellular and mitochondrial ROS levels were measured in SiHa cells following treatment with live* T*.* vaginalis* and* T*.* vaginalis *ESP.

**Figure 1:**
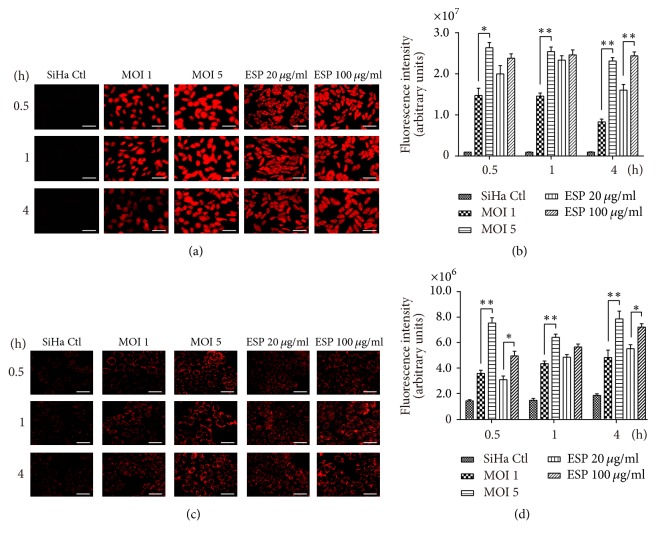
*Trichomonas vaginalis T016 strain induced reactive oxygen species (ROS) production in human cervical mucosal epithelial SiHa cells, in parasite-burden-dependent manner*. SiHa cells were treated with live* T. vaginalis *(multiplicity of infections 1 and 5) and* T. vaginalis *excretory/secretory products (ESP, 20 and 100 *μ*g/mL) at indicated time points. (a and b) Intracellular ROS production was detected by measurement of dihydroethidium (DHE) fluorescence. Intracellular ROS levels were significantly increased in a* T. vaginalis* parasite-burden-dependent manner. (c and d) Mitochondrial ROS production was detected by MitoSOX reagent. Mitochondrial ROS production was significantly increased in parasite-burden- and ESP concentration-dependent manner. The experiment was repeated three times with similar results. Scale bars: 100 *μ*m; ^*∗*^ *P* < 0.05, ^*∗∗*^ *P* < 0.01.

Intracellular ROS levels were prominently increased in SiHa cells treated with live* T. vaginalis* and* T. vaginalis* ESP from 0.5 h after treatment. Intracellular ROS levels were significantly higher in cells treated with* T. vaginalis* MOI 5 than those* T. vaginalis* MOI 1 from 0.5 h until 4 h posttreatment. In cells treated with* T. vaginalis *ESP, intracellular ROS levels were increased in ESP-concentration-dependent manner at 4 h after treatment, but not at 05 and 1 h (Figures 1(a) and 1(b)). Similarly, mitochondrial ROS were also produced after 0.5 h of incubation with live* T*.* vaginalis* and* T*.* vaginalis *ESP. Mitochondrial ROS production was increased in direct proportion to parasite burden and ESP concentration (Figures 1(c) and 1(d)). These findings suggest that* T*.* vaginalis* is a strong inducer of intracellular and mitochondrial ROS, in parasite-burden-dependent manner, and that* T*.* vaginalis* ESP has a similar effect to live* T*.* vaginalis*.

### 3.2. *T*.* vaginalis* Induced Apoptosis through an Intrinsic Mitochondrial Apoptotic Pathway in SiHa Cells

**Figure 2:**
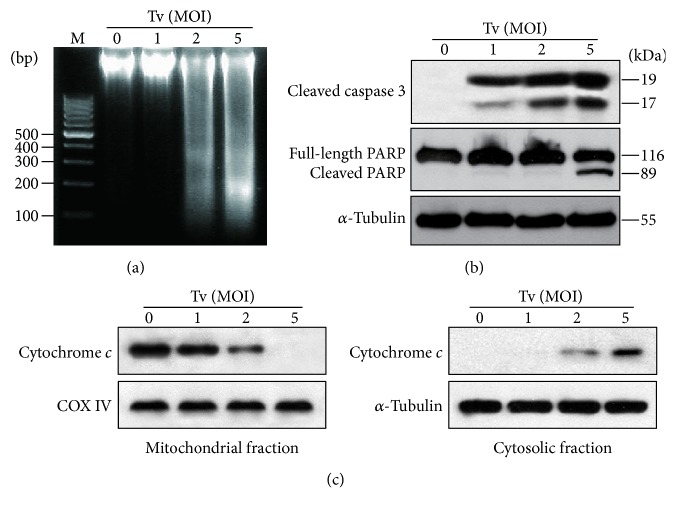
*Mitochondrial apoptosis was induced in SiHa cells following treatment with live T. vaginalis*. (a)* T. vaginalis* induced DNA fragmentation, which is an indicator of apoptosis. (b)* T. vaginalis* induced the cleavage of caspase 3 and PARP. (c)* T. vaginalis* induced cytochrome* c* release from mitochondria into cytosol in parasite-burden-dependent manner. The quality of the fraction experiments was confirmed by assessing the presence of COX IV for the mitochondrial fraction and *α*-tubulin for cytosol fraction. The experiment was repeated three times with similar results. M: marker (100 bp DNA ladder); Tv:* Trichomonas vaginalis*.

We aimed to identify the pathway involved in induction of apoptosis in SiHa cells. SiHa cells were treated with live* T*.* vaginalis *at MOIs of 1, 2, and 5 for 4 h. Live* T*.* vaginalis *induced nucleosomal DNA fragmentation at MOI 2 and 5, in a parasite-burden-dependent manner (Figure 2(a)). Cells treated with live* T*.* vaginalis* also produced cleaved forms of caspase 3 in a parasite-burden-dependent manner and dramatically cleaved PARP at MOI 5 (Figure 2(b)).

Additionally, we examined the release of cytochrome* c* from the mitochondria as an indicator of involvement of the intrinsic apoptotic pathway. High levels of cytochrome* c *were present in the mitochondrial fraction of untreated control cells, but not in the cytosolic fraction. In* T. vaginalis*-treated cells, cytochrome* c* levels were decreased in mitochondria in a parasite-burden-dependent manner, whereas cytosolic cytochrome* c* was increased (Figure 2(c)). These results suggest that* T. vaginalis* induces cell death in cervical mucosal epithelium through the intrinsic apoptotic pathway.

### 3.3. *T. vaginalis* Initially Induced Transient Activation of NF-*κ*B by in SiHa Cells, Followed by a Decline in NF-*κ*B Activation

**Figure 3:**
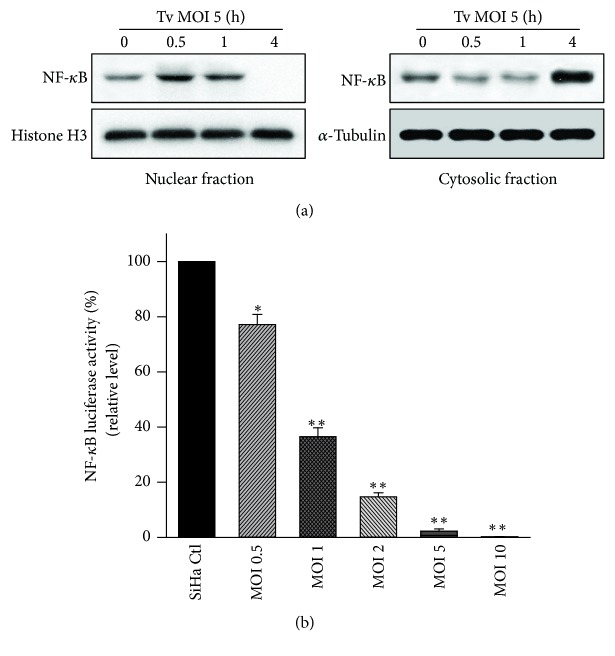
*T. vaginalis initially activated NF-κB in SiHa cells, followed by a decline in NF-κB levels*. (a)* T. vaginalis *transiently induced NF-*κ*B p65 nuclear translocation in SiHa cells; this was followed by a dramatic decline at 4 h after treatment. (b) NF-*κ*B activity of* T. vaginalis*-treated SiHa cells were significantly reduced in a parasite-dose-dependent manner at 4 h posttreatment. The experiment was repeated three times with similar results.^*∗*^ *P* < 0.05 versus SiHa cell control, ^*∗∗*^ *P* < 0.01 versus SiHa cell control.

The NF-*κ*B signaling pathway plays an important role in regulating apoptosis [24]. We used western blotting and luciferase reporter assay to evaluate whether* T. vaginalis* activates the NF-*κ*B signaling pathway in SiHa cells. In SiHa cells treated with* T. vaginalis* at MOI 5, nuclear NF-*κ*B p65 levels were transiently increased until 1 h posttreatment. Thereafter, the nuclear NF-*κ*B p65 level dramatically dropped at 4 h after treatment. In contrast, cytosol NF-*κ*B p65 levels decreased until 1 h after treatment with* T. vaginalis* and then prominently increased at 4 h after treatment (Figure 3(a)). Further, the NF-*κ*B luciferase reporter assay showed a parasite-dose-dependent reduction in NF-*κ*B activity in* T. vaginalis*-treated SiHa cells at 4 h posttreatment. NF-*κ*B activity at* T. vaginalis* MOI 5 and MOI 10 was almost totally suppressed (Figure 3(b)). These results indicate that* T. vaginalis* prevents NF-*κ*B p65 translocation to the nucleus and suppresses NF-*κ*B activity in SiHa cells in a parasite-burden-dependent manner.

### 3.4. The ROS Scavenger, NAC, Blocked* T*.* vaginalis-*Induced Apoptosis and NF-*κ*B Suppression in SiHa Cells

To block ROS production in SiHa cells after treatment with* T. vaginalis*, we used N-acetyl-L-cysteine (NAC) as a ROS scavenger. Before using NAC, we determined its effect on the viability of SiHa cells using an MTT assay. Treatment of SiHa cells with 0.1–1 mM NAC elicited no significant differences in cell viability compared with those in the medium-treated group. SiHa cells treated with 10 mM NAC showed slightly reduced cell viability (Supplemental Figure 1).

**Figure 4:**
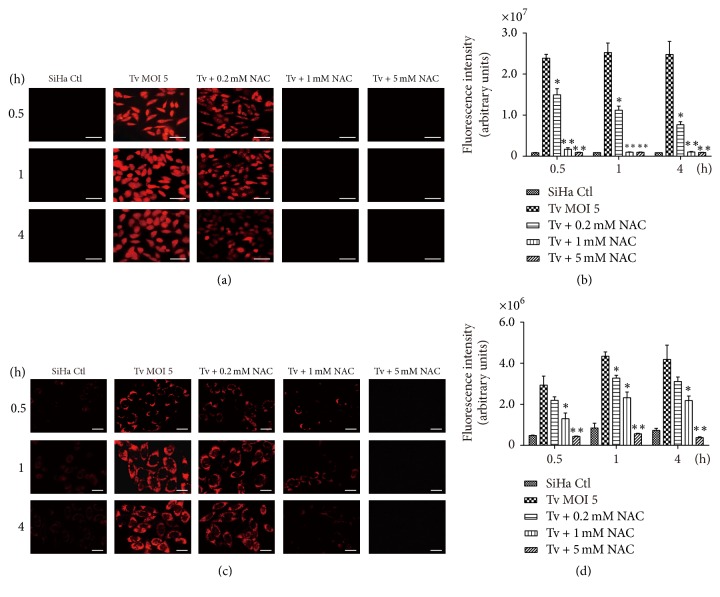
*ROS production was blocked in T. vaginalis-treated SiHa cells after pretreatment with N-acetyl-L-cysteine (NAC)*. (a and b) Intracellular ROS production was detected by dihydroethidium (DHE) fluorescence. Intracellular ROS levels were concentration-dependently inhibited in* T*.* vaginalis*-treated SiHa cells following NAC pretreatment. (c and d) Mitochondrial ROS production was detected by MitoSOX reagent. Mitochondrial ROS production was concentration-dependently prevented in* T*.* vaginalis*-treated SiHa cells following NAC pretreatment. The experiment was repeated three times with similar results. Scale bars: 100 *μ*m; ^*∗*^ *P* < 0.05 versus SiHa cell control, ^*∗∗*^ *P* < 0.01 versus SiHa cell control.

**Figure 5:**
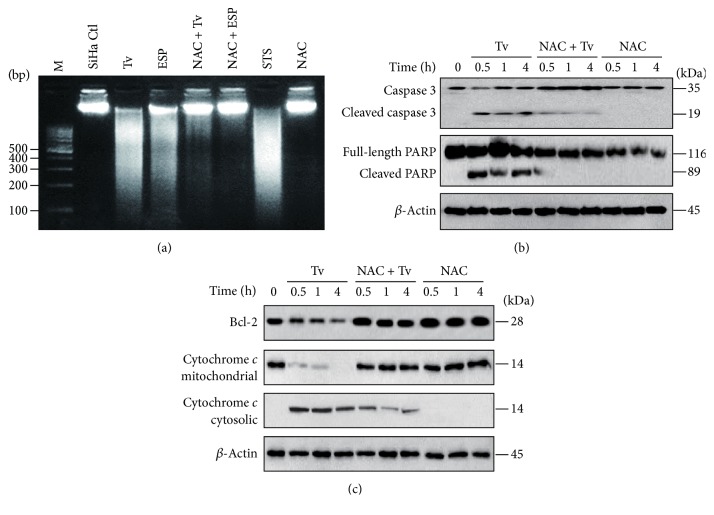
*Apoptosis was prevented in T. vaginalis-treated SiHa cells after pretreatment with NAC*. (a) DNA fragmentation was blocked in* T. vaginalis*-treated SiHa cells by pretreatment with NAC. Lane 1, DNA marker; lane 2, SiHa cell control, lane 3, live* T. vaginalis* (MOI 5); lane 4,* T. vaginalis* ESP (100 *μ*g/mL); lane 5,* T. vaginalis* (MOI 5) + NAC (1 mM); lane 6,* T. vaginalis* ESP (100 *μ*g/mL) + NAC (1 mM); lane 7, staurosporine (STS, 1 *μ*M); lane 8, NAC (1 mM). (b)* T. vaginalis-*induced caspase 3 activation and PARP cleavage in SiHa cells were prevented by NAC pretreatment. (c)* T. vaginalis-*induced mitochondrial cytochrome* c* release of SiHa cells was prevented by NAC pretreatment. The experiment was repeated three times with similar results.

To determine whether ROS are involved in the induction of apoptosis and inactivation of NF-*κ*B in* T. vaginalis*-treated SiHa cells, cells were pretreated with NAC, and then incubated with live* T. vaginalis*. Both intracellular and mitochondrial ROS production were inhibited in* T*.* vaginalis*-treated SiHa cells following NAC pretreatment, in a concentration-dependent manner (Figures 4(a)–4(d)). To examine the relationship between ROS production and apoptosis, DNA fragmentation, caspase activation, cytochrome* c* release, and Bcl-2 levels were evaluated in* T. vaginalis*-treated SiHa cells following pretreatment with NAC. As shown in Figure 5(a), NAC pretreatment strongly suppressed DNA fragmentation induced by* T*.* vaginalis* and* T. vaginalis* ESP. Similarly, in NAC-pretreated SiHa cells,* T*.* vaginalis*-induced cleavage of caspase 3 and PARP were dramatically suppressed (Figure 5(b)). As shown in Figure 5(c), NAC pretreatment significantly increased Bcl-2 protein levels in* T*.* vaginalis*-treated SiHa cells. In addition, cytochrome* c* levels in the mitochondrial fraction was also increased in NAC-pretreated* T. vaginalis*-treated cells, whereas cytochrome* c* levels were decreased in the cytosolic fraction (Figure 5(c)). These results suggest that ROS induced by* T. vaginalis* causes oxidative damage to host cell DNA and that ROS act upstream of the signaling molecules such as cytochrome* c*, caspase 3, and PARP during the induction of apoptosis.

**Figure 6:**
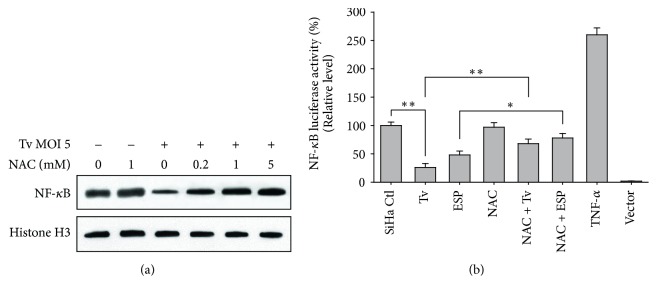
*NF-κB activity significantly increased in T. vaginalis-interacted SiHa cells after pretreatment with NAC*. (a) Nuclear NF-*κ*B p65 levels were significantly increased in* T*.* vaginalis*-treated SiHa cells after NAC pretreatment. (b) NAC pretreatment significantly increased the NF-*κ*B activity attenuated by* T. vaginalis* and* T vaginalis* ESP. Results are expressed as means ± standard deviation (SD). The experiment was repeated three times with similar results. ^*∗∗*^ *P* < 0.01, ^*∗*^ *P* < 0.05.

We also determined whether* T*.* vaginalis-*induced ROS regulate NF-*κ*B activity of SiHa cells. Following pretreatment with NAC, reduced nuclear NF-*κ*B p65 protein levels by* T*.* vaginalis* were significantly increased in a NAC-concentration-dependent manner (Figure 6(a)). In addition, NAC pretreatment was also significantly increased the NF-*κ*B activities in SiHa cells attenuated by live* T. vaginalis* and* T. vaginalis* ESP (Figure 6(b)). These results suggest that ROS production is integral to the NF-*κ*B signaling pathway; thus* T. vaginalis*-induced NF-*κ*B suppression is negated by the suppression of ROS production.

## 4. Discussion

The aim of this study was to determine whether* T. vaginalis-*induced cell apoptosis is mediated by ROS and NF-*κ*B pathway in SiHa cells. Our results show that* T*.* vaginalis* induces intracellular and mitochondrial ROS generation, which causes cytochrome* c *release into the cytosol, activation of a mitochondria-dependent caspase 3, downregulation of Bcl-2, DNA damage, and, finally, commitment of cells to apoptosis. In addition,* T. vaginalis* initially induced translocation of NF-*κ*B p65 into nucleus and then blocked these processes at 4 h after treatment. NF-*κ*B activity also decreased in a parasite-load-dependent manner at 4 h after treatment. However, NAC significantly inhibited* T. vaginalis*-induced nuclear damage, cytochrome* c* release into the cytoplasm, and activation of cleaved caspase 3 and PARP. Moreover, NAC pretreatment also increased NF-*κ*B p65 nuclear translocation in a concentration-dependent manner. Thus, our findings indicate that* T*.* vaginalis* induces SiHa apoptosis in an ROS-dependent manner through NF-*κ*B suppression.

*T*.* vaginalis, *which is known to induce host cell apoptosis in various cell types [5–12], employs various strategies for inducing apoptosis in host cells. Previous reports demonstrated that* T. vaginalis* isolate KT-4 induces apoptosis in human neutrophils by reducing Mcl-1 of Bcl-2 family proteins [25], and* T. vaginalis* induces apoptosis in RAW246.7 murine monocyte/macrophage cells via downregulation of Bcl-xL [6].* T. vaginalis* isolate T016 cleaves Bcl-xL and Mcl-1 in MOI-dependent manner and induces apoptosis in SiHa cells through the dissociation of Bcl-xL/Bim and Mcl-1/Bim complexes [12]. These reports indicate that Mcl-1, Bcl-xL, and Bcl-2 are all of the members of Bcl-1 family proteins and are well-known inhibitor of apoptosis. During the progress of identifying mitochondrial apoptotic component, each lab was using different targets to excavate noble Bcl-2 family proteins, and that is why there are many reports about different proteins from different cell systems. In the present study,* T*.* vaginalis* downregulated Bcl-2 levels, cytochrome* c *release into the cytosol, and activation of caspase 3. Thus, our results also clearly implicate the involvement of the mitochondria-dependent intrinsic apoptotic pathway in* T. vaginalis*-induced SiHa cell death.

It was demonstrated that* T. vaginalis* induces mitochondrial apoptosis through various apoptotic signaling pathways [6, 12, 25]. However, it is poorly understood what trigger molecules are involved in inducing mitochondrial apoptosis of host cells after treatment with* T. vaginalis*.* T. vaginalis* secretes several hydrolytic enzymes; thus some authors demonstrated that* T. vaginalis* cysteine proteases induce apoptosis of primary human vaginal epithelial cells [10] and* T. vaginalis* metalloproteinase induces apoptosis of SiHa cells [12]. Besides the role of* T. vaginalis-*originated signal in inducing apoptosis of host cells, in addition, ROS and the resulting oxidative stress also play a pivotal role in apoptosis [26]. However, the role of ROS in the apoptosis of human cervical mucosal epithelial cells following stimulation with* T. vaginalis* has not been clarified. In the present study,* T. vaginalis* increased the intracellular and extracellular ROS levels in SiHa cells, and led to apoptosis. Furthermore, treatment with the ROS scavenger, NAC, prevented* T. vaginalis*-mediated apoptosis and modulated the activity of the NF-*κ*B pathway. These results suggest that ROS-dependent caspase 3 activation plays an important role in apoptosis of human cervical mucosal epithelial cells by* T. vaginalis*. Our results are similar to the results of previous studies showing that* T*.* vaginalis* causes neutrophil apoptosis via NADPH oxidase-derived intracellular ROS generation [9].

Next, we were eager to understand the role of ROS in relation to the NK-*κ*B pathway for apoptosis induction in* T*.* vaginalis*-treated SiHa cells. To resolve this, we adopted two complementary methods: the detection of NF-*κ*B activity by luciferase reporter assay and NF-*κ*B p65 levels from subcellular components by western blotting. As shown in Figures 3 and 6,* T. vaginalis* inhibited NF-*κ*B p65 nuclear translocation and NF-*κ*B activity in SiHa cells in a parasite-burden-dependent manner. However, NF-*κ*B inactivation was abrogated by pretreatment with ROS scavenger NAC in* T. vaginalis*-treated SiHa cells. Our results suggest that suppression of NF-*κ*B activity in* T. vaginalis*-treated SiHa cells is implicated in ROS generation. The similar results were shown in the previous studies that host cell apoptosis through inactivation of NF-*κ*B activity occurs in human non-small-cell lung cancer cell lines treated with plumbagin [27] and in melanoma A375 cells treated with an extract of* C. anthelminticum* [28]. Surprising, this study showed that NF-*κ*B nuclear translocation occurred at 1 h transiently in* T. vaginalis*-treated SiHa cells and then declined at 4 h posttreatment. Narantsogt et al. [21] also demonstrated that NF-*κ*B translocation occurs within 1 h of* T. vaginalis* adhesion and translocation drops dramatically by 8 h postadhesion. Considering the host-parasite relationship, early activation of NF-*κ*B nuclear translocation may be related to the protection from apoptotic cell death by* T. vaginalis*; subsequently,* T. vaginalis* inhibits NF-*κ*B activity and leads to apoptosis in SiHa cells as a results of failure of NF-*κ*B p50 : p65 heterodimers to translocate to the nucleus. However, whether there is a feedback loop between ROS and NF-*κ*B and the precise regulation mechanism should be further investigated.

**Figure 7:**
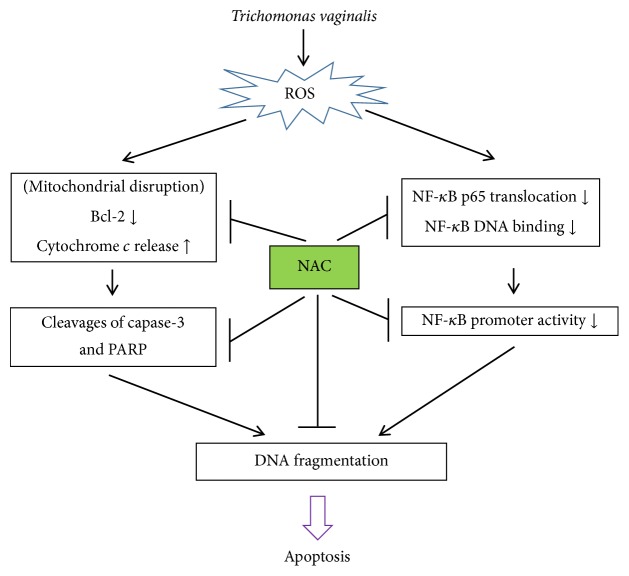
*Schematic diagram of the T. vaginalis-induced apoptotic pathway in cervical mucosal epithelial SiHa cells. T. vaginalis* induces intracellular and mitochondrial ROS production in cervical mucosal epithelial SiHa cells, causing cytochrome c release from the mitochondria and activation of caspase 3. Activation of caspase molecules leads to nuclear fragmentation.* T. vaginalis* also suppresses NF-*κ*B nuclear translocation and activity and initiates apoptosis in cervical mucosal epithelial cells.

In this study, we demonstrated that* T*.* vaginalis *induces apoptosis in human cervical mucosal epithelial SiHa cells through an NF-*κ*B-regulated, mitochondria-mediated pathway involving the activation of ROS (Figure 7). The limitation of the current study may be overcome in future investigations by evaluating primary human vaginal epithelial cells (HVECs). Our findings provide novel insights into the pathophysiology of trichomoniasis and mechanisms of action of ROS in the human cervical mucosal epithelium.

## Supplementary Materials

Supplementary Materials Suppl.  1. Effects of ROS scavenger on the viability of human cervical mucosal epithelial SiHa cells. SiHa cells treated with 10 mM ROS scavenger, N-acetyl-L-cysteine (NAC), showed slightly reduced cell viability. Data are shown as the mean ± standard deviation of results for three different experimental wells.
